# Of PCOS Symptoms, Hirsutism Has the Most Significant Impact on the Quality of Life of Iranian Women

**DOI:** 10.1371/journal.pone.0123608

**Published:** 2015-04-15

**Authors:** Mahnaz Bahri Khomami, Fahimeh Ramezani Tehrani, Somayeh Hashemi, Maryam Farahmand, Fereidoun Azizi

**Affiliations:** 1 Reproductive Endocrinology Research Center, Research Institute for Endocrine Sciences, Shahid Beheshti University of Medical Sciences, 24 Parvaneh, Yaman Street, Velenjak, P.O.Box:19395–4763, Tehran, Iran; 2 Endocrine Research Center, Research Institute for Endocrine Sciences, Shahid Beheshti University of Medical Sciences, 24 Parvaneh, Yaman Street, Velenjak, P.O.Box:19395–4763, Tehran, Iran; Sudbury Regional Hospital, CANADA

## Abstract

**Introduction:**

Polycystic ovary syndrome is a common endocrine disorder affecting women both physically and psychologically and can lead to a poor quality of life compared to their normal counterparts. The aim of the present study was to assess the impact of various clinical features of polycystic ovary syndrome on the health-related quality of life of Iranian women diagnosed with this syndrome.

**Materials and Methods:**

A total of 796 women diagnosed with polycystic ovary syndrome, aged 15–49 years, completed the questionnaires, interviews, and medical assessments required for this study. A reliable and validated Persian version of the health-related quality of life questionnaire for polycystic ovary syndrome patients was used. Linear regression models were used to assess the association between the symptoms of polycystic ovary syndrome and health-related quality of life.

**Results:**

The mean age of participants was 28.02 years. 35.4% of the subjects were classified as overweight or obese. Hirsutism, was reported to have the strongest impact on the patients’ health-related quality of life, followed in descending order by body mass index, irregular menses and infertility. The relative level of hirsutism was directly proportional to decrease in health-related quality of life score (p<0.001).

**Conclusions:**

The results of the study found that hirsutism had the strongest impact on the health-related quality of life measures in Iranian women diagnosed with polycystic ovary syndrome. Health care officials need to evaluate in depth the effect of each clinical feature of polycystic ovary syndrome separately and design management strategies, keeping in mind the psychological and physical manifestations.

## Introduction

Polycystic ovary syndrome (PCOS), a common complex disorder among reproductive-aged women, characterized with hyperandrogenism, ovulatory dysfunction and polycystic ovary morphology [1–3], affects 8–18% of women during their reproductive years [4]. Although the exact pathogenesis of PCOS has remained a mystery [1, 5–7], it is considered as a polygenic trait that is likely caused by the interaction of genetic and environmental factors [7–10].

In addition to irregular menses, hirsutism and infertility, women with PCOS may display a number of metabolic and cardiovascular abnormalities [3, 11–13] and several psychological disorders such as depression, anxiety, marital and social problems and sexual functioning impairment [14–16]. While the underlying causes of these non-reproductive health-related complications are mainly unknown [17], they do negatively affect the quality of life (QoL) of women diagnosed with PCOS [7]. Mood disorders, low sexual satisfaction, weight gain, acne, hair loss, pain, infertility and menstrual irregularity have all been mentioned as factors which decrease QoL of women suffering from PCOS [17, 18]. It has also been shown that lifestyle management strategies to manage these factors can improve the QoL of affected women [10, 18–22].

It is highly recommended that assessment of women with PCOS include not only reproductive and metabolic assessment, but also their health-related QoL (HRQoL) assessment [18]. HRQoL refers to the “physical, psychological and social domains of health seen as distinct areas that are influenced by a person’s experiences, beliefs, expectations and perceptions” [1, 10, 21, 22]. The diminished QoL often faced by women diagnosed with PCOS may be the result of symptoms that are currently causing issues as well as the fear of possible disorders in future [20]. It is not clear which aspects of PCOS have the strongest influence on HRQoL in affected women although negative impacts have been shown to result from hirsutism, acne, hyperandrogenism, metabolic disturbances, menstrual irregularity, obesity and infertility [14, 23, 24]. Further, it is likely that various traditions, cultural-gender identity, religions and ethnicity influence the impact of these factors on HRQoL of women affected by PCOS in various societies [6, 25]. Therefore, the goal of this study was to assess the impact of various clinical features of PCOS on the HRQoL of Iranian women, affected by this syndrome.

## Materials and Methods

### Study subjects and the sampling method

A total of 1035 women diagnosed with PCOS (based on the Rotterdam criteria), aged 15–49 years and referred by gynecologists to the Reproductive Endocrinology Research Center, were recruited to participate in the present study. Based on criteria derived from the 2003 ESHRE/ASRM (Rotterdam Criteria) PCOS is diagnosed as the presence of at least two of three of the following: 1) Oligo/anovulation (AnOvu), 2) hyperandrogenism (HA), and 3) Polycystic ovaries (PCO) [26]. The statistical power of this sample size was calculated according to these parameters: P = 0.085, α = 0.95, d = 0.025, cluster design effect = 2 and a non-response rate = 0.15.

Those women who were diagnosed with pituitary disease, adrenal, thyroid or metabolic disease (n = 174), or who had been previously diagnosed as psychiatric, and/or were using psychiatric medicines, except women with a self-reported history of mood disorders, were excluded (n = 31), as were those who had missing data on any of the variables evaluated (n = 34). We did not exclude single women, as infertility is a concern not only among women who want to be pregnant, but also among adolescent girls suffering from PCOS [18].

During face-to-face interviews, a standard questionnaire was completed. The questionnaire included demographic information and reproductive status (emphasizing the regularity of menstrual cycles), gynecological history, hyperandrogenic symptoms, family history of irregular menstrual cycles and hirsutism. Written consents were obtained after the purpose and procedure of the study were thoroughly explained to the participants or to the guardians for participants, aged <18 years. Hirsutism was assessed using the mFG scoring method by the interviewer (a trained staff member of the research center) and a gynecologist who supervised the process.

Patients completed a reliable, validated Persian version of a standard questionnaire, “Health-related Quality of Life (HRQoL) Questionnaire for PCOS (PCOSQ)”, which is a 26 item, multi-dimensional, self report questionnaire for the quality of life assessment among PCOS women. Its validity and reliability has been demonstrated previously [27, 28].

The PCOSQ evaluates five domains of the patients’ quality of life, i.e. emotions, body hair, weight, infertility problems and menstrual problems [29]. Patients were asked to choose the response option that best suited their feelings during the past 2 weeks. Scores ranged 1–7 for each item in which, the higher score represents better function. By adding the scores of the individual items that comprise the domain and dividing by the number of questions in that domain, individual domain scores were obtained. There were 8 questions related to emotions, 5 each for body hair and weight, and 4 each for the infertility and menstrual problem domains. The total score was obtained by adding the five domain scores.

All participants underwent clinical examinations including body weight, height, waist (WC), hip circumferences (HC) and blood pressure. The body mass index (BMI) was calculated as the subjects’ weight in kilograms divided by their height in meters squared (kg/m2).

In the present study oligo/anovulation (AnOvu) was defined as <10 and/or >14 menstrual cycles per year and/or if there was amenorrhea. Clinical hyperandrogenism was defined as a hirsutism score >7 according to a modified Ferriman-Gallway (mFG) evaluation [26, 30]. Acne and androgenic alopecia are less useful clinical markers of hyperandrogenism [31], which can occur in both PCOS-affected and unaffected populations [32], and were therefore excluded as independent markers of hyperandrogenism. Polycystic ovary manifestation (PCO) using ultrasonography was defined based on the presence of 12 or more follicles with a 2–9 mm diameter and/or increased ovarian volume (10 cm^3^) [12, 33, 34].

#### Statistical analysis

Continuous variables are expressed as the mean ± standard deviation (SD) and/or the median and interquartile (IQ) ranges, as appropriate. The categorical variables are expressed as percentages. To select important PCOS symptoms which affect HRQoL, we used the “ENTER” method in linear regression models adjusted for age, BMI, education (below diploma, diploma and higher), occupation (being paid or not) and patients’ chief complaint (with at least one PCOS symptom or without any symptom). The results were presented as β amounts for association between HRQoL and its domains scores and continuous variables and for mean differences in scores of categorical variables. We did not include women whose PCOS diagnostic laboratory assessments had been done in different laboratories with different laboratory kits and only used the results obtained from the same kits in our statistical analysis. Data analysis was performed using the SPSS 15.0 PC package (SPSS Inc., Chicago, IL), with statistical significance set at P<0.05.

#### Ethics Statement

The ethical review board of the Research Institute for Endocrine Sciences approved the study proposal and written informed consent was obtained from all subjects or from guardians for those aged <18 years.

## Results

Of 1035 women, 796 ones met all eligibility criteria for final inclusion the study. The mean±SD age of participants was 28.02±6.02 years. Most women (86.4%) reported an education level with or above the diploma level. Only 26.6% of the participants had a job with an income. In many cases irregularity of menstrual cycles was one of the first reasons for seeking treatment (57.8%). Mean menarchal age was 13.14±1.56 years. Mean BMI was 26.63±5.70 kg/m^2^; 20.3% and 15.1% of the participating women were overweight and obese, respectively. The characteristics of PCOS women according to their main perceived manifestations are presented in Table 1.

**Table 1:** Characteristics of PCOS women according to their main perceived manifestations.

	All participants (n = 796)	Hyperandrogenism (n = 304)	AnOvu (n = 611)	Infertility (n = 120)
**Age (years)** ^✵^	28.02 ± 5.70	27.31 ± 5.70	27.65 ± 6.00	30.07 ± 5.15
**BMI (kg/m** ^**2**^ **)** ^✵^	26.63 ± 5.70	27.48 ± 5.79	26.80 ± 5.91	28.64 ± 4.97
**WC (cm)** ^✵^	85.74 ± 13.27	87.07 ± 13.41	85.90 ± 13.37	91.45 ± 12.39
**Education (%)**				
**High School**	13.6	14.1	13.4	23.3
**Diploma**	33.7	33.9	34.5	42.5
**University**	52.7	52	52.1	34.5
**Occupation (%)**				
**Unemployed**	73.49	77.5	74.3	80%
**Employed**	26.51	22.5	22.7	20%
**Chief Complaint (%)**				
**Infertility**	21.1	15.8	17.3	64.2
**Irregular menses**	57.8	38.2	21.8	10.8
**Excess body hair**	21.9	61.2	69.1	35
**Other**	23.0	20.4	19.5	10
**Androgens** ^✵✵^				
**FAI** ^†^	2.60(1.41, 4.62)	2.60(1.42, 4.82)	2.64(1.42, 4.82)	2.40(1.40, 5.00)
**DHEAS (μg/dl)** ^‡^	143.00 (89.69, 219.39)	151.85(89.59, 224.89)	152.18(91.11, 222.38)	143.00(91.00, 213.14)
**A**_**4**_ **(μg/dl)** ^§^	0.16(0.10, 0.24)	0.16(0.10, 0.24)	0.17(0.10, 0.25)	0.22(0.10, 0.50)

^✵^Mean±SD.

^✵✵^Median (IQ_25_, IQ_75_).

^†^(n = 289).

^‡^(n = 233).

^§^(n = 118).

The mean scores for the QoL domains obtained from the questionnaire showed that the lowest score provided by the participants was in the menstrual problem domain and the highest score was in the infertility domain Table 2. The mean scores for each question ranged 1–7.

**Table 2:** Mean scores of 26 items and five PCOSQ domains in the study population.

	Score (mean ± SD)
**Emotion**	5.05 ± 1.41
Depressed as a result of having PCOS?	5.50 ± 1.88
Easily tired?	4.42 ± 2.04
Moody as a result of having PCOS?	5.27 ± 1.93
Had low self-esteem a result of having PCOS?	5.74 ± 1.75
Felt frightened of getting cancer?	5.13 ± 2.10
Worried about having PCOS?	4.97 ± 2.11
Self-conscious as a result of having PCOS?	5.18 ± 2.05
Late menstrual period?	4.17 ± 2.45
**Body Hair**	5.02 ± 1.94
Growth of visible hair on chin?	5.05 ± 2.23
Growth of visible hair on upper lip?	5.51 ± 2.03
Growth of visible hair on your face?	4.86 ± 2.37
Embarrassment about excessive body hair?	4.97 ± 2.36
Growth of visible body hair?	4.72 ± 2.30
**Weight**	4.98 ± 1.88
Concerned about being overweight?	4.36 ± 2.43
Had trouble dealing with your weight?	4.64 ± 2.33
Felt frustration in trying to lose weight?	5.37 ± 2.00
Feel like you are not sexy because of being overweight?	5.74 ± 1.89
Have difficulties staying at your ideal weight?	4.80 ± 2.26
**Infertility Problems**	5.14 ± 1.74
Concerned with infertility problems?	4.75 ± 2.32
Felt afraid of not being able to have children?	4.86 ± 2.24
Feel a lack of control over the situation with PCOS?	5.51 ± 1.95
Feel sad because of infertility problems?	5.44 ± 2.18
**Menstrual Problems**	4.61 ± 1.54
Headaches?	5.46 ± 2.05
Irregular menstrual periods?	3.85 ± 2.44
Abdominal bloating?	4.79 ± 2.21
Menstrual cramps?	4.35 ± 2.19

The questionnaire data was analyzed using a regression model adjusted for age, BMI, education, occupation and chief complaint. This analysis revealed that hirsutism had the strongest association with QoL where a one unit increase in standard deviation of mFG score was associated with a 0.3 decrease in the HRQoL score (p<0.001). This was followed by the associations of QoL with BMI (p<0.001), AnOvu (p<0.001) and infertility (p = 0.002). The HRQoL score was significantly increased by ageing (Table 3).

**Table 3:** Linear regression analysis of possible related factors in HRQoL and domains.

Regression Model	HRQoL	Emotions	Body hair	Weight	Infertility problems	Menstrual problems
**Hirsutism Score** ^†^	-0.37^✵^	-0.26^✵^	-0.62^✵^	-0.15^✵^	-0.09^✵^	-0.17^✵^
**BMI (kg/m** ^**2**^ **)** ^†^	-0.24^✵^	-0.10^✵^	-0.02	-0.56^✵^	-0.11^✵^	-0.06
**AnOvu** ^‡^						
**Yes**	-0.18^✵^	-0.19^✵^	-0.05	-0.13^✵^	-0.02	-0.27^✵^
**No**	Ref^§^					
**Infertility** ^‡^						
**Yes**	-0.11^✵^	-0.09^✵^	-0.02	-0.03	-0.26^✵^	-0.00
**No**	Ref					
**Age (years)** ^†^	0.07^✵^	0.03	0.00	0.07^✵^	0.14^✵^	0.03
**Occupation** ^‡^						
**Yes**	-0.06	-0.01	-0.01	-0.08^✵^	-0.04	-0.09^✵^
**No**	Ref					
**Chief Complaint** ^‡^						
**Yes**	-0.05	-0.04	-0.01	0.02	-0.05	-0.13^✵^
**No**	Ref					
**Education Level** ^‡^						
**≥Diploma**	-0.01	0.00	-0.02	-0.05	0.08^✵^	-0.051
**<Diploma**	Ref					

^✵^p<0.05.

^†^β is reported as association amounts.

^‡^ β is reported as mean differences of scores between two groups.

^§^ Reference group.

The hirsutism score could significantly predict reductions in all domain scores, including body hair, emotions, menstrual problems, weight and infertility problems, in their order of severity (p<0.001). Women who had hirsutism were significantly younger and more obese than those without the condition (p = 0.01 and p = 0.001, respectively).

## Discussion

The present study demonstrated that while HRQoL scores for Iranian women diagnosed with PCOS are negatively affected by various manifestations of PCOS, including hirsutism, obesity, AnOvu and infertility, the most significant association was with hirsutism. A one unit increase in standard deviation of the mFG score was associated with a 0.3 decrease in HRQoL score. The results of our study are consistent with a Sri Lankan study involving 146 PCOS women and 170 controls evaluated using the WHOQoL-BREEF questionnaire where it was reported that the mFG score was the main predictor of psychological distress and was correlated with the emotion domain [35]. Not only is hirsutism itself a major concern for women diagnosed with PCOS, but the time and energy spent at concealing it aggravates the distress further [18]. In a qualitative study, Ekback et al. (2009) evaluated the experiences of women with hirsutism and reported that a majority of these women had a negative self-body image and considered themselves ugly and unattractive, which lowered their self-esteem and limited their social interaction [36]. However, in a study involving 128 American women with PCOS, obesity was the domain of the highest concern, while body hair was the lowest; FG scores were inversely correlated with body hair and the emotion domains, respectively [10]. Two studies conducted in Australia and Germany also found the body weight domain to be the most strongly associated with lower QoL [17, 21]. In a qualitative study involving 15 adolescents with PCOS, weight and body image were found to be significant in reducing HRQoL scores [11]. In Germany, the QoL reported by 120 PCOS women were assessed and the results revealed obesity and hirsutism to be correlated to lower QoL scores, as measured by the SF-36. It seems that in Iran, in addition to symptoms such as obesity and hirsutism, which are related to the patient’s appearance, irregular menstruation and infertility are major factors that contribute to psychiatric problems [23, 24] and hence reduce the HRQoL score. It has been reported that women diagnosed with PCOS consider themselves inferior to their normal counterparts and feel disappointed, depressed, and embarrassed because of their percieved lack of femmininity, i.e. irregular and unpredictable menstrual cycles and reproductive inability [37]. The symptoms of PCOS can be quite variable depending on the patients’ ethnicity [38]. The impact of these symptoms on the extent of the decrease in the HRQoL scores is probably dependent on ethnicity and socio-cultural factors [1, 6, 11]. In western countries, obesity is perceived as a negative and abnormal feature [20], while in many Eastern women, obesity, as opposed to being lean, symbolizes affluence/prosperity, indicating socio-cultural differences [35, 39]. Visible symptoms of PCOS largely result in emotional and social functioning issues [11]. It seems that in our study population, the patients’ current symptoms were of greater concern than the possibility of future ones.

Symptoms of hyperandrogenism are often considered to be a result of poor hygiene or diet. The current data shows that the social stigma associated with hirsutism and acne, two symptoms of hyperandrogenism, increases anxiety [10, 11]. We also found that BMI, a possible indicator of the quality of the patients’ diet, was the second most important contributor to a decreased HRQoL score. Patients who suffer from hirsutism and obesity may not be able to find a partner [24]; women who have supportive partners, despite the manifestations of PCOS, have better emotional well-being [1]. Although BMI affects both psychological and physical dimensions of SF-36, it has more impact on the former [1]. It has been shown that a loss in body weight of only 5% could significantly improve PCOS clinical manifestations even if the women were still not of normal weight [7]; however exercise itself has not been confirmed to reduce the concerns associated with PCOS [4, 21].

Our findings demonstrated that any change in AnOvu score was associated with a decrease in HRQoL score; a one unit change from the standard deviation in AnOvu, whether less or more, decreased the HRQoL score by 0.18. Our participants got the lowest QoL score in the menstrual problem domain, similar to the findings of Hahn et al (2005), who also reported that irregular menstrual cycles were annoying for the patients [23]. Patients may perceive menstrual irregularity as a primary reason of infertility and hirsutism, which, in turn, results in increased stress, that worsens symptoms leading to more severe ovulatory impairments.

According to our results, infertility also significantly decreased the QoL, although this was reported as the lowest concern for our patients’ QoL. This is similar to a study involving Sri Lankan women diagnosed with PCOS where concerns regarding their infertility had a smaller impact of QoL [35]. Contrary to these findings, a study comparing Austrians and Muslim immigrants found infertility to be the major concern reported by both groups [6]. The extent to which infertility can affect QoL is highly dependent on its duration and history of treatment in addition to cultural, ethnic and social factors [23]. It seems that due to advanced treatment options in Iran, although these women are hopeful for AnOvu and infertility treatment, their anxiety and concerns about hirsutism remain; currently a common option is the permanent reduction of hair growth using laser-assisted hair removal, which is the most effective on women with high contrast between skin and hair color [40], a combination rarely seen among Iranian women who are not very fair and lack the contrast mentioned; this method is also very expensive [41].

The strengths of the present study include its relatively large sample size, as most previous studies are limited by small sample sizes, of around 100 women [20], and our use of regression analysis after adjustment for potential confounding variables as other studies mostly used correlations to assess the impact of symptoms of PCOS on the QoL for women with PCOS. This study does have some limitations; our data on the patients’ hormonal status was not all obtained by the same kits, which is known to have significant effects on the values obtained, and therefore we were only able to statistically analyze a subset of the data that was obtained using a particular kit. Androgen excess has been known as a possible factor in psychological disorders associated with PCOS [22, 42]. In the study by Pastore et al (2011), free testosterone was associated with the severity of depression symptoms among obese PCOS women [22], although this was contrary to study by Hahn et al (2005), which did not find testosterone levels to be correlated with psychological scales [23].

## Conclusions

In conclusion, the QoL of Iranian women with PCOS seems to be affected more by the severity of hirsutism, as compared to other PCOS symptoms. Practitioners should consider that the QoL for women of diverse ethnic backgrounds is affected differently by the various symptoms of PCOS, and that these differences should be taken into account when prioritizing treatment planning and counseling.
